# Deep-RBPPred: Predicting RNA binding proteins in the proteome scale based on deep learning

**DOI:** 10.1038/s41598-018-33654-x

**Published:** 2018-10-15

**Authors:** Jinfang Zheng, Xiaoli Zhang, Xunyi Zhao, Xiaoxue Tong, Xu Hong, Juan Xie, Shiyong Liu

**Affiliations:** School of Physics, Huazhong University of Science and Technology, Wuhan, Hubei 430074 China

## Abstract

RNA binding protein (RBP) plays an important role in cellular processes. Identifying RBPs by computation and experiment are both essential. Recently, an RBP predictor, RBPPred, is proposed in our group to predict RBPs. However, RBPPred is too slow for that it needs to generate PSSM matrix as its feature. Herein, based on the protein feature of RBPPred and Convolutional Neural Network (CNN), we develop a deep learning model called Deep-RBPPred. With the balance and imbalance training set, we obtain Deep-RBPPred-balance and Deep-RBPPred-imbalance models. Deep-RBPPred has three advantages comparing to previous methods. (1) Deep-RBPPred only needs few physicochemical properties based on protein sequences. (2) Deep-RBPPred runs much faster. (3) Deep-RBPPred has a good generalization ability. In the meantime, Deep-RBPPred is still as good as the state-of-the-art method. Testing in A. thaliana, S. cerevisiae and H. sapiens proteomes, MCC values are 0.82 (0.82), 0.65 (0.69) and 0.85 (0.80) for balance model (imbalance model) when the score cutoff is set to 0.5, respectively. In the same testing dataset, different machine learning algorithms (CNN and SVM) are also compared. The results show that CNN-based model can identify more RBPs than SVM-based. In comparing the balance and imbalance model, both CNN-base and SVM-based tend to favor the majority class in the imbalance set. Deep-RBPPred forecasts 280 (balance model) and 265 (imbalance model) of 299 new RBP. The sensitivity of balance model is about 7% higher than the state-of-the-art method. We also apply deep-RBPPred to 30 eukaryotes and 109 bacteria proteomes downloaded from Uniprot to estimate all possible RBPs. The estimating result shows that rates of RBPs in eukaryote proteomes are much higher than bacteria proteomes.

## Introduction

RNA binding proteins (RBPs) play important functions in many cellular processes, such as post-transcriptional gene regulation, RNA subcellular localization and alternative splicing. With significant function in biology, many high-throughput experimental techniques have been developed to identify new RBPs in human, mouse, S. cerevisiae and C. elegans^1–10^. After RBPs have been identified, CLIP-related experimental technologies^11–14^ are applied to reveal the binding sites in RNAs. Also, many computational methods have been proposed to predict interaction of protein with RNA^15–18^ and RBPs^19–25^. RBP predictors can predict the RBPs, and then CLIP-related techniques can further reveal RNAs interacting with these RBPs. However, previous computational methods only considered only part features or known RNA binding domain (RBD) which plays a significant role in RBPs prediction. So, we proposed RBPPred integrating as much as features to address this problem^22^. Benchmarking on datasets shows that RBPPred is better than other approaches. But RBPPred runs slowly because it requires to run blast against a huge protein NR database to generate PSSM matrix. However, the prediction speed is important because a large number of RBPs are still unknown in many species. To overcome this shortcoming, we present Deep-RBPPred which is based on deep learning.

In recently years, deep learning technology has been used in many aspects in bioinformatics and proved as a power tool^26–32^. For predicting protein binding sites in RNA sequence, DeepBind^32^ is the first CNN-based model to predict the binding affinity. Deep-rbp^29^ and iDeep^30,31^ are two deep learning methods which both take RNA structure into consideration. These methods outperform the conventional approaches in term of prediction accuracy. However, deep learning algorithm is still not applied to RBPs prediction. In Deep-RBPPred, we apply a deep convolutional neural network instead of SVM. Since CNN-based method requires to input a fixed length feature vector, two solutions are handled to meet this requirement. The first solution is to pad all the sequences to fixed length sequences, and then one-hot encoding is used to encode the sequences. The second solution is to design the features by hand. It is not appropriate to predict RBPs with the padding solution because the length of RBPs varies over a wide range (50–10 K, see methods). Based on this consideration, we employ the hand-designed features which are proved effective to represent RBPs in RBPPred. Unlike RBPPred, we only employ physicochemical features including hydrophobicity, polarity, normalized van der Waals volume, polarizability, side chain’s charge and polarity. These features are used to train the weights of 11 layers convolutional neural network with Tensorflow^33^. Deep-RBPPred presents comparable results to RBPPred but is significantly more efficient in the testing. And it also only needs few physicochemical features.

## Methods

### Training sets

In order to train our deep learning model, we employed the training set used in the RBPPred. The details of generating the training set have been described in RBPPred^22^. Here we just simply describe the generating process. The positive samples are collected from the Uniprot database^34^, which is retrieval with GO term’RNA binding’ to search this protein database because the Uniprot database includes RBPs with x-ray crystal structures and RBPs identified by high-through experiment. For the negative samples, we took the approach from SPOT-stru^35^. The negative sequences are collected from PDB, by using PISCCES^36^ with sequence identity cutoff 25%, sequence length between 50 and 10,000 and resolution of X-ray better than 3.0 Å. The collected sequences are then mixed together so that the redundant proteins are removed with sequence identity of 25% by psi-cd-hit in the CD-HIT package^37^. Finally, the training set includes 2780 RBPs and 7093 non-RBPs.

The training set consisting of different amount of RBPs and non-RBPs is known as an imbalance training set. The classification algorithm tends to favor the majority class when it is trained in the imbalance dataset. So, we randomly select 2780 non-RBPs to generate the balance dataset together with 2780 RBPs.

### Testing set

For testing our deep learning model, we used the testing set from RBPPred^22^. However, only the identical sequences between the training and the testing set are removed. This may lead to a bias result caused by the redundance between training and testing set. So, we remove the homology sequences by CD-HIT. The testing and training set are mixed together to be clustered by CD-HIT with sequence identity cutoff 30%. Then all the sequences are discarded from testing set if they are in the same cluster with the training sequences. This can ensure the testing sequences are independent with training set. For one cluster, we only select the sequence provided by CD-HIT to ensure a non-redundant testing set. We finally collected 488 sequences including 239 negative samples and 249 positive samples, which are composed of 72 RBPs and 13 non-RBPs for A. thaliana, 129 RBPs and 164 non-RBPs for H. sapiens, 48 RBPs and 62 non-RBPs for S. cerevisiae. Comparing to the previous testing dataset including 2546 sequences, 2058 sequences are discarded for the redundance.

### Protein features and encoding

**Figure 1:**
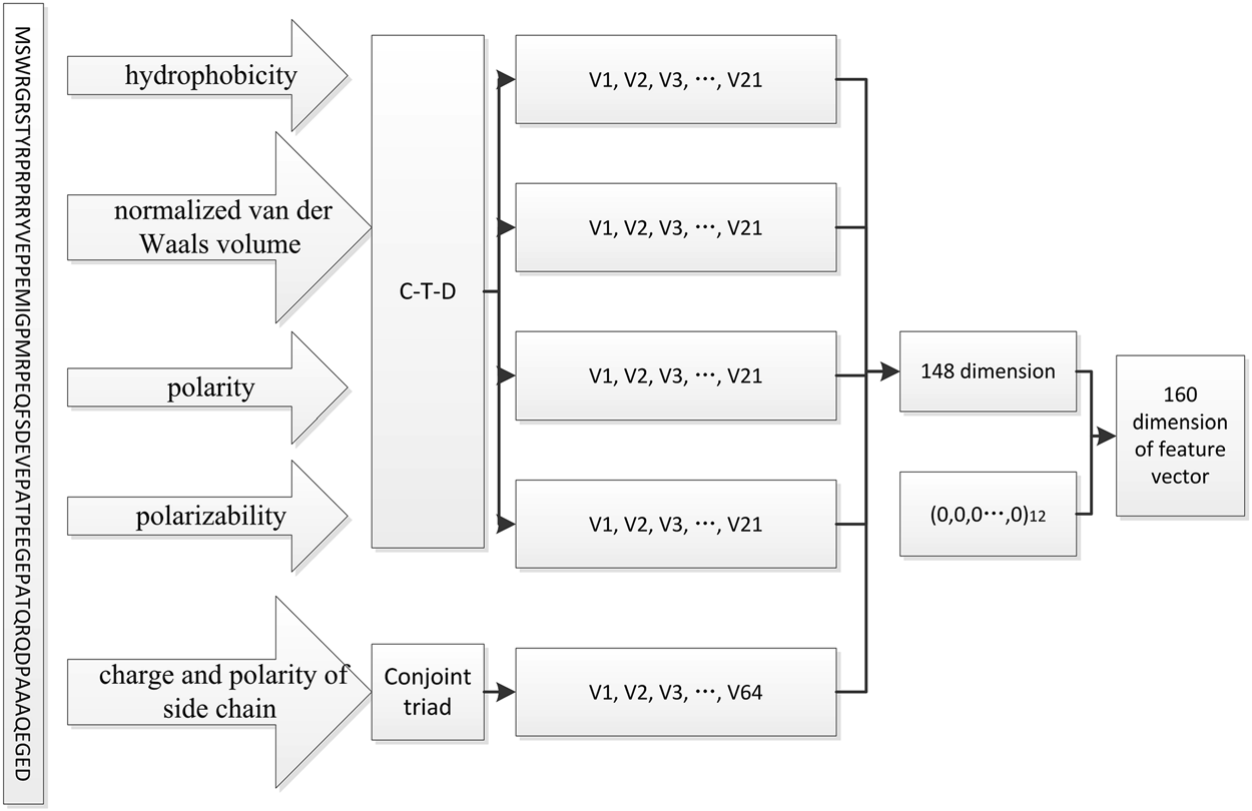
Process of encoding a protein sequence into the 160 dimension feature vector. For the properties of hydrophobicity, polarity, normalized van der Waals volume, polarizability, the global protein sequence descriptors (C-T-D) was employed to encode each feature vector with 21 dimension (v1, v2, v3, v4, …, v21). According to charge and polarity of side chain, the protein sequence was encoded to a vector of 64 dimension (v1, v2, v3, v4, …, v64) through the conjoint triad encoding method. This process is a part of RBPPred encoding process^22^.

The protein is encoded to a feature vector by the approach described in RBPPred^37^. But the evolutionary information and predicted secondary structure are discarded due to the computational time. The solvent accessibility is also discarded. At last, a 148-dimensional vector is encoded to represent each protein sequence including the properties of hydrophobicity, normalized van der Waals volume, polarity and polarizability, charge and polarity of side chain. We expand the dimension of feature vector to 160 with the expanded feature values assigned to 0 due to the CNN network architecture. Finally, we collect a total 160-dimensional feature vector to represent a protein sequence, as shown in Fig. 1. The detail of encoding the physicochemical properties is described in RBPPred^22^. The program encoding the feature vector is extracted from the RBPPred software package.

### Performance evaluation

The performance is evaluated by sensitivity (SN), specificity (SP), accuracy (ACC) and Matthews Correlation Coefficient (MCC) which are defined as following:

$${\rm{Sensitivity}}\,({\rm{SN}})={\rm{TP}}/({\rm{TP}}+{\rm{FN}})$$

$${\rm{Specificity}}\,({\rm{SP}})={\rm{TN}}/({\rm{TN}}+{\rm{FP}})$$

$${\rm{Accuracy}}\,({\rm{ACC}})=({\rm{TP}}+{\rm{TN}})/({\rm{TP}}+{\rm{TN}}+{\rm{FN}}+{\rm{FP}})$$

$$\begin{array}{c}{\rm{Matthews}}\,{\rm{Correlation}}\,{\rm{Coefficient}}\,({\rm{MCC}})=\\ ({{\rm{TP}}}^{\ast }{\rm{TN}}-{{\rm{FP}}}^{\ast }{\rm{FN}})/{\rm{sqrt}}({({\rm{TP}}+{\rm{FN}})}^{\ast }{({\rm{TP}}+{\rm{FP}})}^{\ast }{({\rm{TN}}+{\rm{FP}})}^{\ast }({\rm{TN}}+{\rm{FN}}))\end{array}$$

where, TP is true positive, and FN is false negative. TN refers to true negative and FP refers to false positive. The AUC is also applied to measure the performance.

### Network architecture of Deep-RBPPred

**Figure 2:**
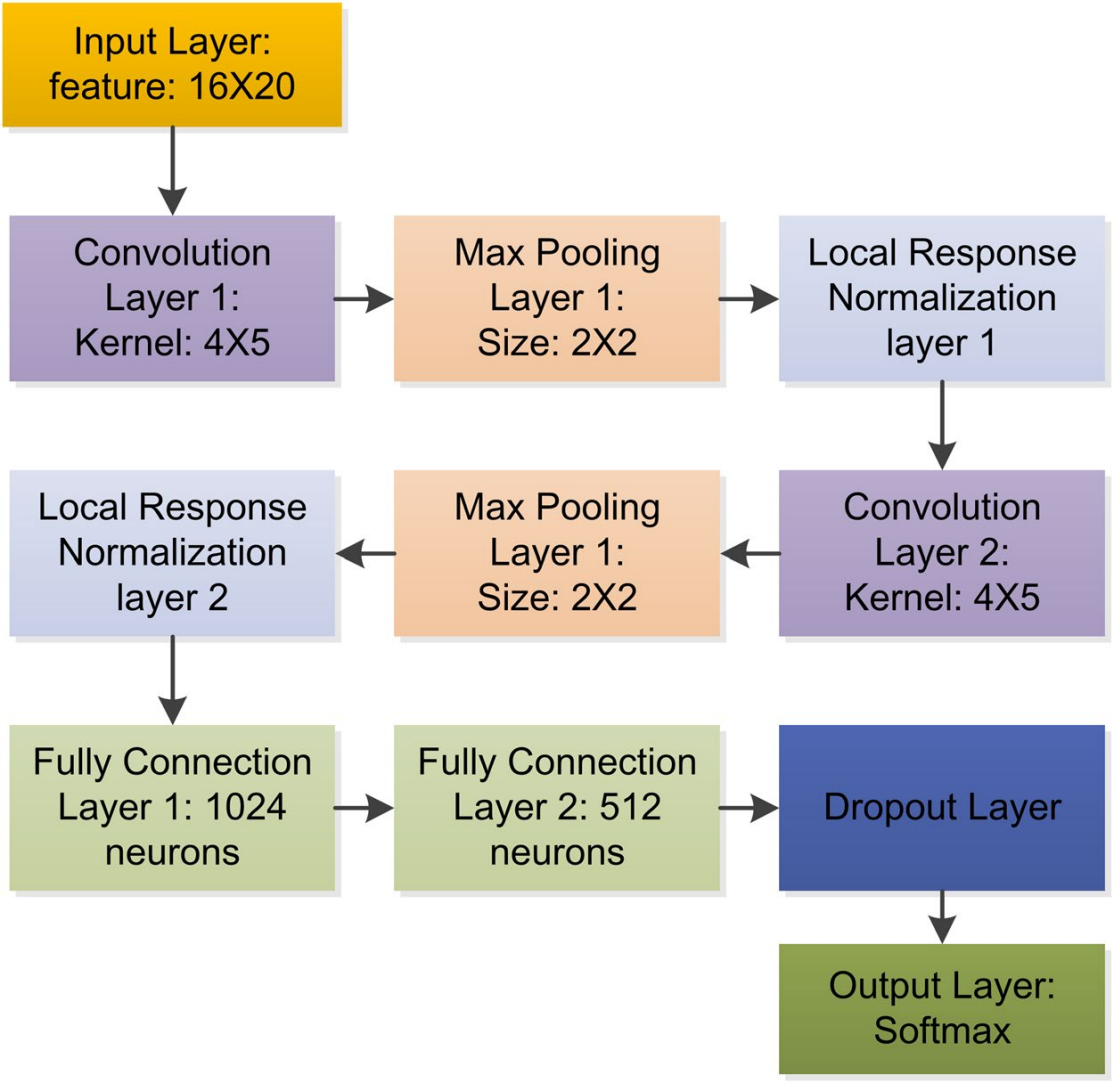
Network architecture of Deep-RBPPred. Deep-RBPPred is a CNN network including 11 layers. Convolution Layer and Max Pooling Layer are designed to automatically process the feature. Local Response Normalization layer and Dropout Layer are designed to avoid over-fitting. Softmax layer is used to classify the protein with probability score. The batch size is assigned to 200. The learning rate is set to 0.0001.

Deep-RBPPred is a Convolutional Neural Network (CNN)^38^ with tensorflow. In Fig. 2, it shows the network architecture of Deep-RBPPred. The protein feature vector is reshaped to a tensor with shape 8 × 20 in order to apply the 2D-convolution function. So the input layer is a size of 8 × 20 feature tensor representing a protein. The following layer is a convolution layer with a kernel size of 2 × 5. In this layer, 32 convolution kernels are set to filter the input features. The third layer is a max pooling layer with a size of 2 × 2. The feature size will be reduced to 4 × 10 after the layer. And the next layer is a local response normalized layer. This layer is set to increase the generalization ability. The following three layers are convolution layer, max pooling layer and local response normalized layer, respectively. Then the feature tensor is flatted to a 640-dimensional vector. The following two layers are fully connected layers with 512 and 256 neurons, respectively. The 10th layer is a dropout layer^39^ which randomly discards some neurons in the training phase. The dropout probability is set to 0.5. The final layer is the Softmax layer which is used to classify RBPs or not. The output of this model is a probability score which describes the probability of an RBP. All the activation functions in neurons are ReLU^40^. All the weights in neurons are added a L2 regularization operation. The L2 regularization losses are added to the final loss function. Adam optimizer is employed to minimize final loss consisting of cross-entropy between the label and probability score and L2 regularization loss of neurons. In this architecture, the number of trainable variable is 480,930. In training process, the learning rate is set to 0.0001.

## Result

### 10-fold cross-validation on the training set

**Figure 3:**
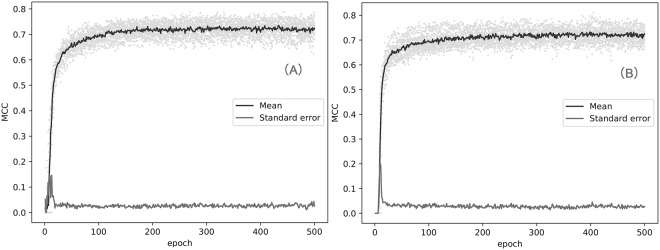
Training process of 10-fold cross-validation on balance set (**A**) and imbalance set (**B**). In 10-fold cross-validation, we calculate the mean MCC of each epoch and the standard error of MCC. For balance set, the highest average MCC is 0.74 and the highest standard error is 0.15. For imbalance set, the highest average MCC is 0.73 and the highest standard error is 0.20.

To avoid the overfitting and estimate an appropriate epoch of our models in the whole training sets, we perform the 10-fold cross-validation on the balance and imbalance training set. As shown in Fig. 3, the balance model converges between 100 and 200 epochs. The imbalance model converges between 300 and 400 epochs. Comparing to the balance model, the imbalance converges at a later epoch. This may be caused by the more sequences in the imbalance training set. Figure 3 also shows that the balance model achieves a slighter higher MCC than the imbalance model. The result of 10-fold cross-validation indicates that 500 epochs can be used in the training process and avoid the overfitting caused by a higher epoch. The result of 10-fold cross-validation also indicates that the parameters of network (batch size, the number of neuron, learning rate) can work in this model.

### Training process and model selection

**Figure 4:**
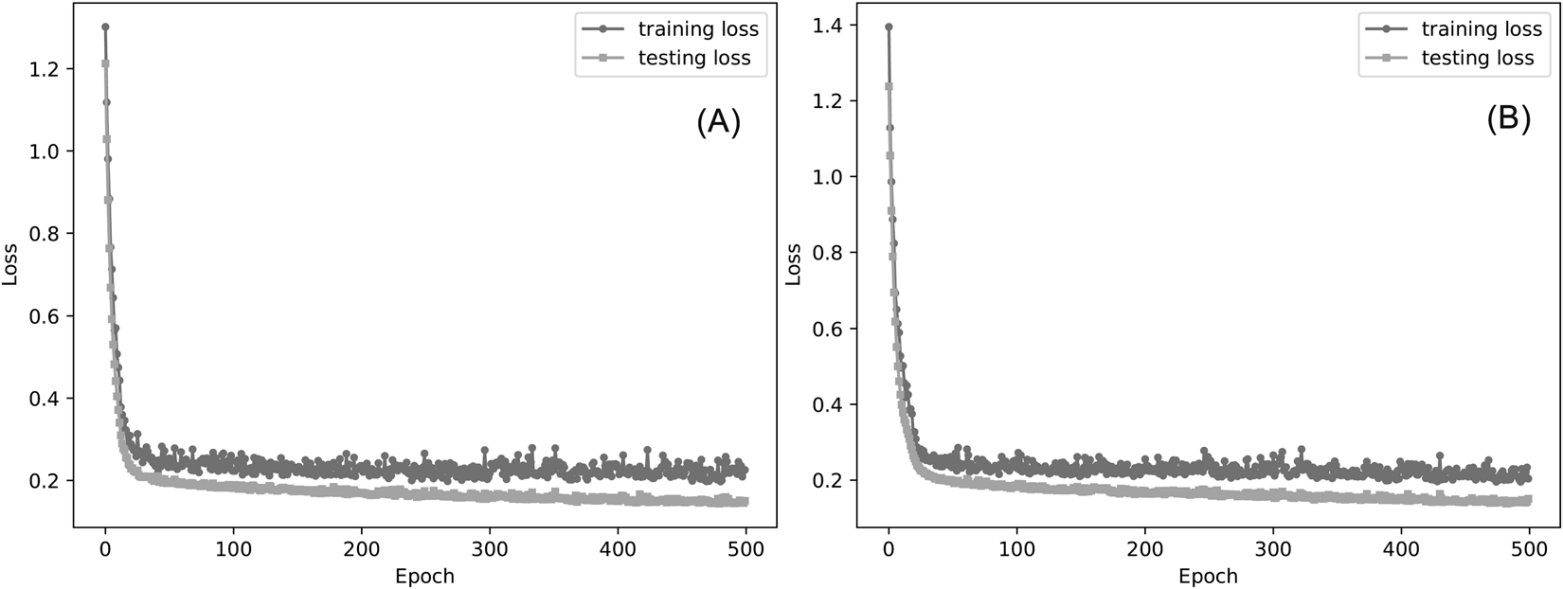
The training process in imbalance (**A**) and balance training set (**B**). The loss is defined as the sum of L2 regularization loss and the cross entropy (see text).

In order to achieve models constructed on the whole training sets, the CNN network is trained in the balance and imbalance training set with the epoch determined in the 10-fold cross-validation. The training and testing loss are used to evaluate the models in each epoch. As shown in Fig. 4, the training and testing loss decrease with the epoch. This training processes are similar to the 10-fold cross-validation (Fig. S1). The testing loss decreases rapidly at the early epoch and then the value remains the same after the convergence point. In theory, all models near the convergence point can be used as the final model. In order to get the best prediction performance, the balance model of 390^th^ epoch and imbalance model of 242^th^ epoch are selected as final models. The training loss of final balance/imbalance model is 0.15/0.17, which is almost equal to loss of the 10-folds validation process (Fig. S1). The testing loss of final balance/imbalance is 0.23/0.24. This indicates that our models is not overfitting.

### Performance in independent testing and comparison to other models

**Figure 5:**
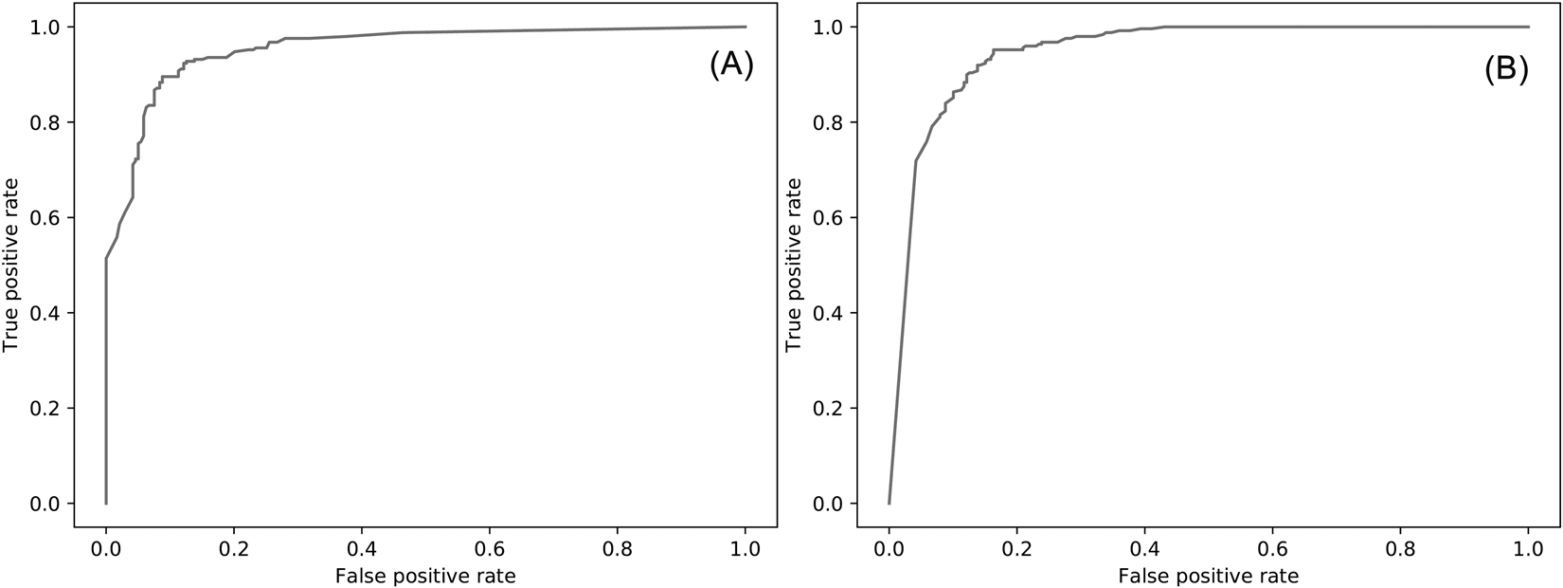
ROC for Deep-RBPPred-imbalance (**A**) and Deep-RBPPred-balance (**B**). The AUC for balance/imbalance model is 0.95/0.95.

In this section, we comprehensively evaluate our deep learning models in a non-redundant testing dataset. Firstly, we compare the power of two of the most popular machine learning algorithms, SVM and CNN, in RBPs prediction. Secondly, the balance and imbalance models are compared to reveal the affections of these two models in predicting RBPs. Thirdly, three RBPs prediction approaches, SONAR^25^, RNApred^23^ and RBPPred^27^ are compared with our deep learning models in a non-redundant testing dataset. All results are shown in Tables 1–3 and Fig. 5. The ROC curves are plotted in Figs S2 and S3.

**Table 1:** Performance on the testing set for the SVM-based model.

Model	SVM-imbalance			SVM-balance		
Dataset	S	H	A	S	H	A
ACC	0.75	0.80	0.71	0.76	0.74	0.85
SN	0.56	0.64	0.65	0.83	0.81	0.83
SP	0.90	0.93	1.0	0.71	0.70	0.92
AUC	0.86	0.88	0.93	0.85	0.85	0.92
MCC	0.50	0.60	0.47	0.54	0.50	0.60

^*^H, S, and A stand for H. sapiens, S. cerevisiae, and A. thaliana species respectively.

**Table 2:** Performance comparison on the testing set.

Model	Deep-RBPPred-balance			Deep-RBPPred-imbalance			RBPPred		
Dataset	H	S	A	H	S	A	H	S	A
ACC	0.91	0.81	0.95	0.91	0.85	0.94	0.91	0.88	0.90
SN	0.96	0.94	0.94	0.89	0.83	0.94	0.85	0.85	0.88
SP	0.87	0.71	1.0	0.93	0.85	0.92	0.96	0.90	1.0
AUC	0.97	0.90	0.99	0.96	0.91	0.98	0.98	0.95	0.98
MCC	0.83	0.65	0.85	0.82	0.69	0.80	0.81	0.76	0.72

^*^H, S, and A stand for H. sapiens, S. cerevisiae, and A. thaliana species respectively.

**Table 3:** Performance of RNApred in testing dataset.

Method	RNApred			SONAR
Dataset	S	H	A	H
ACC	0.65	0.68	0.86	0.88
SN	0.90	0.88	0.93	0.92
SP	0.45	0.52	0.46	0.85
AUC	0.82	0.80	0.83	0.84
MCC	0.38	0.41	0.42	0.77

^*^H, S, and A stand for H. sapiens, S. cerevisiae, and A. thaliana species respectively. SONAR is developed for the human, and gene name is used as input (not protein sequence). The gene name may be the same between different species, so we only test the performance in human proteome.

In order to make a comparison of the power of machine learning algorithm in predicting RBPs, we also train a balance model and an imbalance model with SVM. The SVM-based models are constructed on the training sets with the libsvm-3.22^41^ and tested in the testing dataset. The results are shown in Table 1. As shown, the SVM-imbalance model achieves MCC values of 0.50, 0.60 and 0.47 for S. cerevisiae, H. sapiens and A. thaliana. The SVM-balance model achieves MCC values of 0.54, 0.50 and 0.60 for S. cerevisiae, H. sapiens and A. thaliana. The result indicates there is no significant difference for the imbalance and balance model. In Table 2, Deep-RBPPred-balance achieves MCC values of 0.82 for H. sapiens, 0.69 for S. cerevisiae, 0.80 for A. thaliana, which are both much higher than the balance and imbalance model of SVM.

For non-RBPs in the testing set, the SVM-balance model obtains an average SP of 0.78 ((0.71 + 0.70 + 0.92)/3), which is much better than average SP of 0.62 ((0.56 + 0.64 + 0.65)/3) for the imbalance model. For predicting RBPs in the testing dataset, the SVM-imbalance model achieves an average of SN 0.94 ((0.90 + 0.93 + 1.0)/3), which is much higher than average SN of 0.82 ((0.83 + 0.81 + 0.83)/3) for the balance model. Indeed, the SVM models trained on the balance or imbalance model have an effect on the non-RBPs prediction. That is, the balance model has a better/worse predicting ability in RBPs/non-RBPs than the imbalance model. This effect is not significant in CNN when comparing with SVM. As shown in Table 2, Deep-RBPPred-balance achieves an average SN of 0.95 ((0.96 + 0.94 + 0.94)/3). For non-RBPs, Deep-RBPPred-balance achieves an average SP of 0.86 ((0.87 + 0.71 + 1.0)/3), which is lower than average SP of 0.90 ((0.93 + 0.85 + 0.92)/3) of Deep-RBPPred-imbalance. Figure 5 shows the ROC of Deep-RBPPred.

Deep-RBPPred is also tested and compared to other models on the testing set. Total three predictors are compared. The first approach is RBPPred, which is developed by our group previously. The second approach is RNApred which employees the amino acid composition or PSSM to predict RBPs. The third method is SONAR which integrates protein-protein interaction network and other features to predict the RBPs. The result of SPOT-Seq-RNA is not shown here because it has been compared with RBPPred^22^.

As shown in Table 2, Deep-RBPPRed-balance achieves MCC values of 0.83, 0.65 and 0.85 for H. sapiens, S. cerevisiae, A. thaliana, respectively. The performance of the imbalance model of Deep-RBPPred is almost as good as the balance model. RBPPred achieves MCC values of 0.81, 0.76 and 0.72 for H. sapiens, S. cerevisiae, A. thaliana respectively. We can find that RBPPred and Deep-RBPPred have different performances in S. cerevisiae and A. thaliana proteomes. The average MCC of Deep-RBPPred-balance ((0.83 + 0.65 + 0.85)/3) has a value almost as high as RBPPred ((0.81 + 0.76 + 0.72)/3). As shown in Table 3, Deep-RBPPred achieves a much higher MCC than RNApred ((0.38 + 0.41 + 0.42)/3). Deep-RBPPred also performs better than SONAR in the human proteome.

### Capacity of prediction new RBPs

To test the predicting ability of Deep-RBPPred on new RBPs, we collect 299 new RBPs created between 2015-05-24 (consistent with RBPPred) and 2017-09-27 from Uniprot. In this section, only the RBPPred is compared because that the RBPPred have been proved to have better predicting ability than other methods^22^. 280 and 265 of 299 new RBPs are correctly predicted by Deep-RBPPred-balance and Deep-RBPPred-imbalance, but only 260 RBPs are predicted by RBPPred^22^. Deep-RBPPred performs better than RBPPred. One protein (Uniprotid: P0DOC6) can’t be calculated by RBPPred for that no protein sequences can be found by Blast. We also collect the 130 experimently determined human RBPs published in Wen and the co-workers’ work^42^. RBPPred correctly predicts 24 of 130 RBPs, while Deep-RBPPred-imbalance and Deep-RBPPred-balance correctly predict 63 and 92 RBPs respectively. These results indicate that Deep-RBPPRed has better predicting ability than RBPPred.

### Computational time

Running time is an important metric to measure a model. We list the computational time of Deep-RBPPred in Table 4. The table shows that Deep-RBPPred is a very fast RBP predictor. Here we do not list the computational time of RBPPred because it costs much more computational time. Comparing with RBPPred, Deep-RBPPred predicts RBPs without using blast to generate PSSM matrix which is a time-consuming step. Take the advantage of computational time, Deep-RBPPred can be used to estimate RBPs in proteome scale quickly.

**Table 4:** Computational time of Deep-RBPPred running in Centos with Intel(R) Xeon(R) CPU E5-2620 v2 @ 2.10 GHz and GeForce GTX 1080Ti.

Proteome	UP000000559	UP000005640	UP000000589	UP000006548
Times (CPU)	4 s	40 s	33 s	30 s
Times (GPU)	3 s	14 s	13 s	12 s

^*^UP000000559, UP000005640, UP000000589 and UP000006548 include 1000, 20231, 16946 and 15524 protein sequences. Reviewed proteomes are downloaded from the Uniprot.

### RBPs estimation in the 139 reviewed proteomes

Deep-RBPPred is applied to estimate the RBPs in 139 reviewed proteomes for 109 bacteria and 30 eukaryote species. There are two problems in the Uniprot proteome dataset. Firstly, reviewed and un-reviewed sequences are both included in the Uniprot. The un-reviewed sequence may not be a real protein. So, the reviewed proteomes are used in the prediction. The second problem is that almost all reviewed proteomes are incomplete. For example, truepera radiovictrix (proteome id: UP000000379) only contained one reviewed sequence. These two problems can result in a bias prediction. In order to select some appropriate proteomes, we filter the proteomes with the number of sequences. For eukaryote species, the amount is set to 1/10 of S. cerevisiae. For bacteria, the number is set to 1/10 of *E. coli*. Finally, we filter out 109 bacteria and 30 eukaryote proteomes from all the bacteria and eukaryote proteomes.

**Figure 6:**
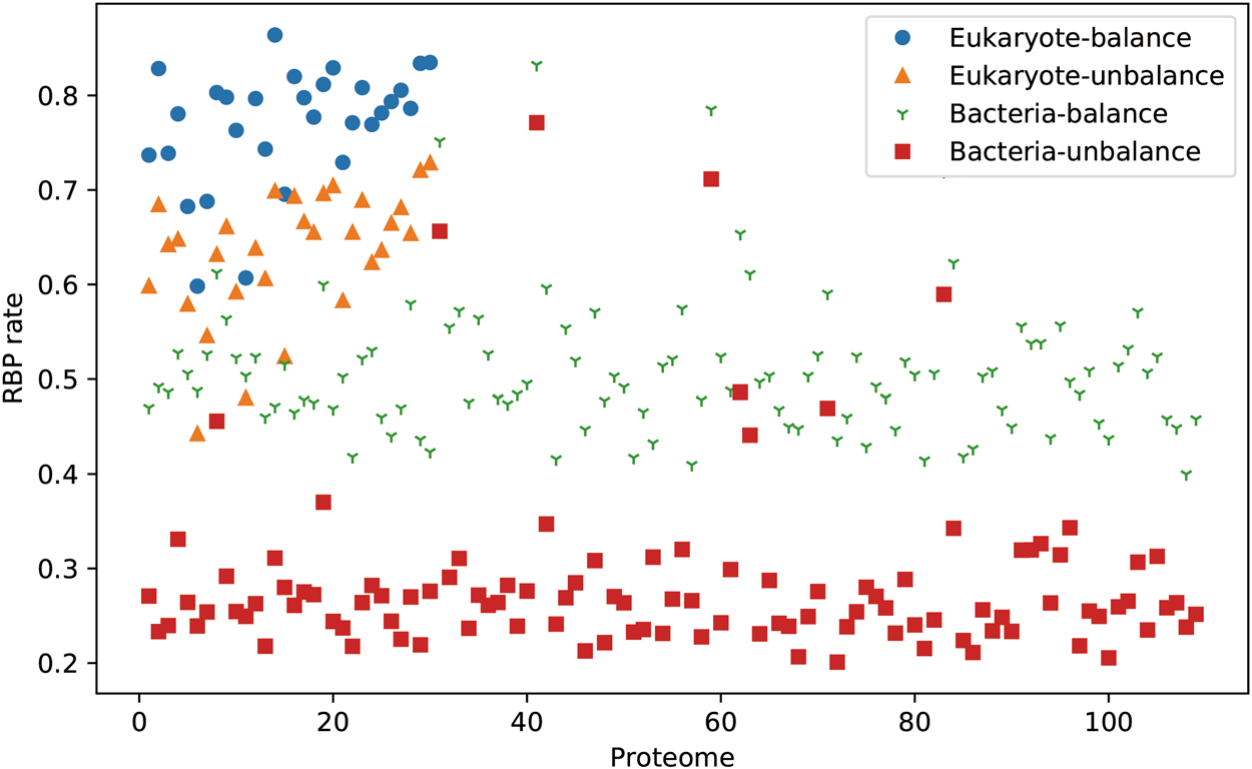
The RBPs rate estimation of Deep-RBPPred in the reviewed eukaryota and bacteria proteomes from uniprot. Total 109 bacteria and 30 eukaryote proteome are kept due to the limitation of sequence amount in the proteome (1/10 of yeast for eukaryote and 1/10 of *E. coli* for bacteria). The labels ‘Eukaryote-balance’ stands for that the eukaryotes proteome is predicted by the balance model.

The results of prediction are shown in Fig. 6. The balance model predicts more RBPs than the imbalance model in bacteria and eukaryote. For the predicting results with the balance model and imbalance model, we found an interesting phenomenon that the rate of RBPs in eukaryotes proteome is higher than bacteria. This result implies RBPs may function in more complex cellular processes in eukaryotes. For the human proteome, we estimate 14,744 RBPs with the imbalance model.

## Discussion

In this study, we develop two RBPs predicting models (the balance and imbalance model) based on CNN which only need hydrophobicity, normalized van der waals volume, polarity and polarizability, charge and polarity of side chain of protein sequence. Comparing with SVM models, we show that the CNN-based model performs better than SVM-based model. In comparing the balance and imbalance model, both the CNN-based and SVM-based classification show a preference for the major class. The result from the testing dataset shows that our deep learning models perform as good as RBPPred which is the best model so far. More importantly, Deep-RBPPred needs fewer features than RBPPred. Deep-RBPPred was then applied to estimate RBPs in 139 reviewed proteomes from the Uniprot dataset. The result shows that the RBPs rate in the bacteria is smaller than the eukaryote proteome. Deep-RBPPred-imbalance predicts 14,744 RBPs for the whole reviewed human proteomes. This number is almost 10-fold more than the number of the RBPs identified by high through experiments. This may be caused by these high throughput experimental limitations. For example, “Interactome Capture” only identifies the RBPs which bind to mRNA^2^. It may lose the RBPs binding to the non-coding RNA.

In general, deep learning methods are applied in a large-scale data. A classic application of CNN-based methods is to classify the image. In this application, data augmentation approaches are used to enlarge the number of samples. And a large dataset may reduce the risk of deep learning model in overfitting. In Deep-RBPPred, we remove the redundant sequences as other researches have done. The process can be regarded as the opposite of data augmentation process in image recognition. In addition, L2 regularization and dropout layer^43^ are added to avoid overfitting in the architecture of our deep learning. The process of 10-cross validation (Fig. 3) shows the MCC is almost no longer increases after 100^th^ epoch. The process of training (Fig. 4) also shows the training/testing loss does not change too much round 0.2/0.14. These phenomena imply Deep-RBPPred is not overfitting. The real number of RBPs is still unknown and new RBPs are discovered as time goes by. Our prediction may benefit the RBP community.

## Electronic supplementary material

Supplementary Information

## Data Availability

Deep-RBPPred is written in the python, availability as an open source tool at http://www.rnabinding.com/Deep_RBPPred/Deep-RBPPred.html.
