# Supplementary material for: Deep-RBPPred: Predicting RNA binding proteins in the proteome scale based on deep learning
Source: Sci Rep. 2018 Oct 15;8:15264. doi: 10.1038/s41598-018-33654-x (PMC6189057; doi:10.1038/s41598-018-33654-x)
Supplement: Supplementary file 1 — Supplementary Information [file 41598_2018_33654_MOESM1_ESM.pdf]

Supplementary Information for

Deep-RBPPred: Predicting RNA binding proteins in the proteome scale based on deep learning

Jinfang Zheng, Xiaoli Zhang, Xunyi Zhao, Xiaoxue Tong, Xu Hong, Juan Xie and Shiyong Liu*

School of Physics, Huazhong University of Science and Technology, Wuhan, Hubei 430074, China

* To whom correspondence should be addressed. Email: liushiyong@gmail.com

This file includes Figure S1, Figure S2 and Figure S3.

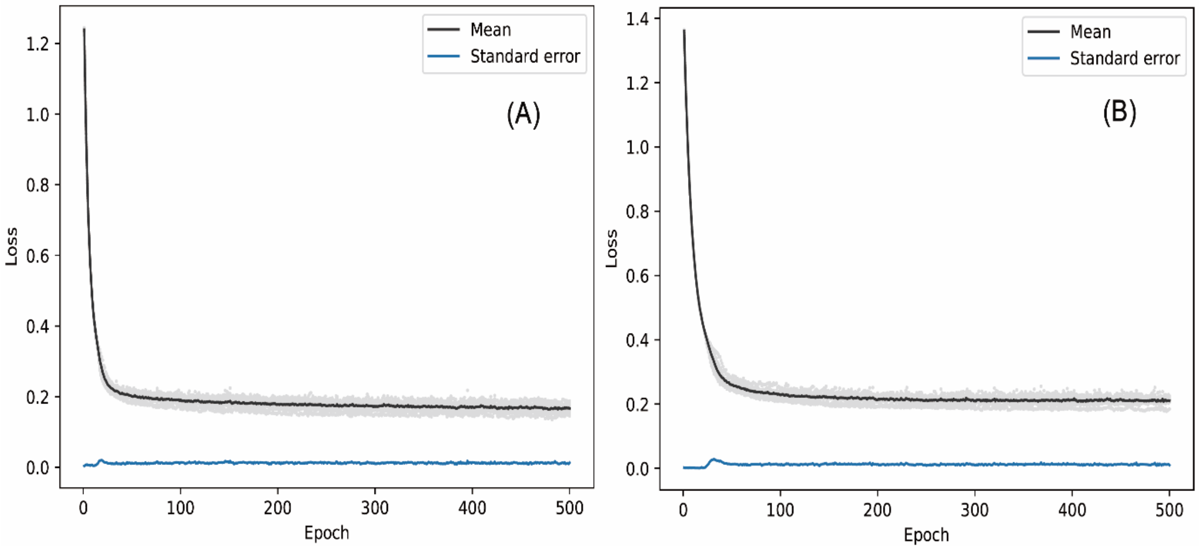

Fig. S1. The process of 10-fold cross-validation. The average loss is plot against epoch in the imbalance set (A) and balance set (B). For the imbalance set, the minimum loss is 0.14. For the balance set the minimum loss is 0.18.

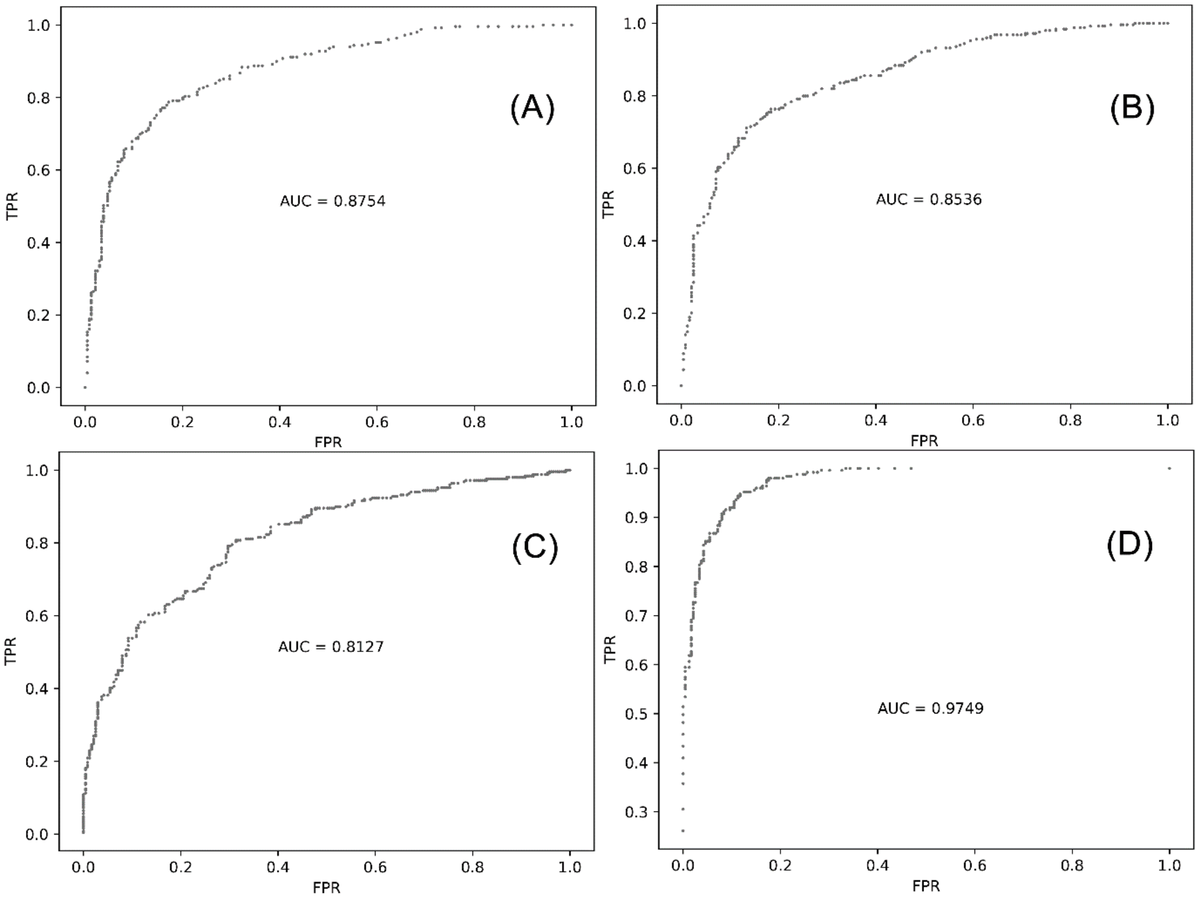

Fig. S2. ROC for SVM-imbalance (A), SVM-balance (B), RNAPred (C) and RBPPred (D) in the testing dataset, respectively. The AUC are 0.85, 0.88.0.81 and 0.95, respectively.

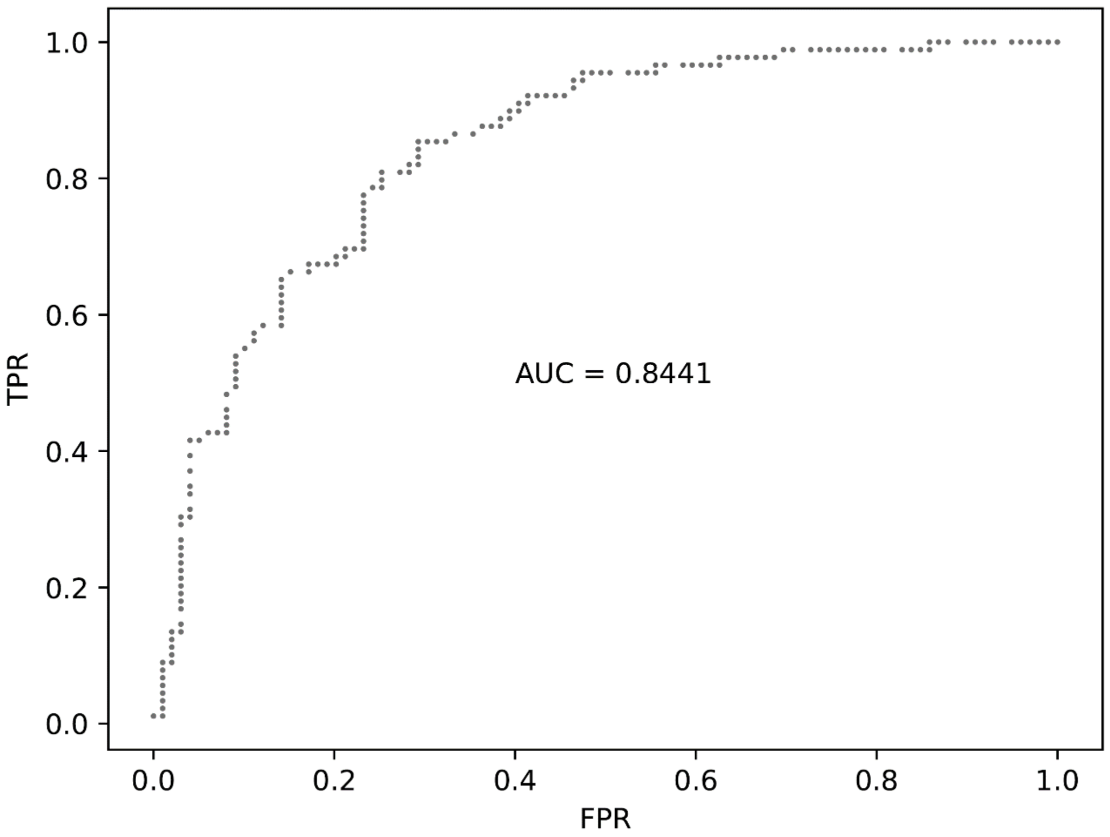

Fig. S3. ROC for SONAR in the testing dataset. The AUC of SONAR is 0.84.
